# Plant Size Plays an Important Role in Plant Responses to Low Water Availability and Defoliation in Two Woody Leguminosae Species

**DOI:** 10.3389/fpls.2021.643143

**Published:** 2021-04-09

**Authors:** Ning Wang, Qiang Li, Xiao Liu, Shijie Yi, Mingming Zhao, Xinke Sun, Huijia Song, Xiqiang Peng, Peixian Fan, Qun Gao, Yongtao Wang, Linqian Yu, Hui Wang, Ning Du, Renqing Wang

**Affiliations:** ^1^School of Life Sciences, Institute of Ecology and Biodiversity, Shandong University, Qingdao, China; ^2^Shandong Provincial Engineering and Technology Research Center for Vegetation Ecology, Shandong University, Qingdao, China; ^3^Qingdao Forest Ecology Research Station of National Forestry and Grassland Administration, Shandong University, Qingdao, China; ^4^Qingdao Forestry Station, Qingdao, China

**Keywords:** artificial defoliation, carbon storage, drought, stress tolerance, recovery

## Abstract

Plant size influences plant responses to combined environmental factors under climate change. However, their roles in plant ecophysiological responses are not fully understood. Two rapidly growing Leguminosae species (*Robinia pseudoacacia* and *Amorpha fruticosa*) were used to examine plant responses to combined drought and defoliation treatments (two levels of both treatments). Both 1.5 month-old seedlings and 3 month-old seedlings were grown in a greenhouse, and seedling growth, leaf gas exchanges, stem hydraulics, and concentrations of non-structural carbohydrates were determined after 60 days of treatment. Our results indicated defoliation had no significant effect on plant height, basal diameter, and total biomass whatever plant sizes and species. Under the low water availability treatment, the defoliated seedlings significantly increased by 24% in stem water potential compared with non-defoliated seedlings in large *R. pseudoacacia*. Compared with the high water availability in large non-defoliated *R. pseudoacacia* seedlings, the low water availability significantly reduced by 26% in stem starch concentration to maintain the stem soluble sugar concentration stable, but not in small *R. pseudoacacia* seedlings. We also found a negative correlation between leaf and root soluble sugar concentration under low water availability in *A. fruticosa.* The results demonstrate defoliation could relieve the effect of low water availability in large seedlings. Large seedlings had more compensatory mechanisms in response to defoliation and drought treatments than small seedlings, thus species with large carbon reserves are more recommended for vegetation restoration under combined drought and defoliation conditions. Future studies with more species are crucial for obtaining more rigorous conclusions.

## Introduction

Global climate change is expected to increase both the level of insect damage and the occurrence of severe drought (Jacquet et al., 2014; Nahar et al., 2017; Gely et al., 2020). Consequently, in natural environments, trees are often subjected to combined biotic and abiotic (environmental) stress (Myers and Kitajima, 2007; Kulkarni and De Laender, 2017; Peck and Mittler, 2020). In addition, plant size plays a very important role in plant growth and physiological activities (Rosas et al., 2013). However, there are few studies focused on the combined effects of defoliation, drought, and plant size on plant growth (Quentin et al., 2012; Jacquet et al., 2014), especially in terms of their response mechanisms at the individual level. Studying the effects of plant size on the response and intrinsic mechanisms of tree species to combined insect disturbances and varying water availability is extremely important for predicting tree species’ growth and dynamics in the context of climate change, and it could provide more comprehensive information on current changes in forest ecosystem productivity (Jacquet et al., 2014; Assal et al., 2016; Wagg et al., 2017).

Among all abiotic factors influenced by climate change, drought is the primary factor limiting plant growth in many forest ecosystems (Falcão et al., 2017). Climate change has been predicted to lead to changes in global rainfall and rainfall distribution patterns (Tietjen et al., 2017), causing the redistribution of water resources in time and space; the number, intensity, and duration of droughts will increase, especially in the areas where drought was already a problem (IPCC, 2014). Drought will not only limit plant growth and productivity (Choi et al., 2002; Sperlich et al., 2016; Wu and Su, 2016), but it will also alter carbon allocation (Ivanov et al., 2019). Plants reduced stomatal aperture under drought to save water and prevent water transport from failure (Gieger and Thomas, 2002; Ohashi et al., 2006; Chen et al., 2010). However, stomatal closure under drought conditions is likely to reduce carbon uptake at the same time, and the carbon balance may become negative (Quentin et al., 2012). Non-structural carbohydrates (NSCs) are critical to maintain plant metabolism under drought condition. If the assimilation is reduced, the deficiency of NSCs will affect plant growth, respiration and other metabolic processes (Dietze et al., 2014).

Many woody species are frequently attacked by insect herbivores. Recovery from insect defoliation is vital for plant growth, especially in the context of climate change. Compensatory growth of individual plants after defoliation has been predicted; however, no hypotheses are universally accepted (Barry et al., 2012). In general, after defoliation, individual plants could recover by increasing water transport capacity, up-regulating photosynthesis, and enhancing leaf biomass ratio (Turnbull et al., 2007; Quentin et al., 2012). However, some studies found that defoliation treatments did not result in the up-regulation of leaf photosynthesis in remaining leaves (Wiley et al., 2013). As a result, previous studies have not found a consistent perspective regarding whether defoliation promotes or inhibits the growth and development of trees (Karolewski et al., 2010). The extent and timing of individual plant recovery after leaf removal is related to the removal frequency and species identity (Jacquet et al., 2014).

It is important to deepen our understanding of the effects of drought on the ability of trees to recover from insect attacks (Gaylord et al., 2013). A previous study suggested that gas exchange after defoliation depends on soil water availability (Quentin et al., 2012). Defoliation reduces the total transpiration area and improves water retention in the remaining leaves (Quentin et al., 2011), even though one study showed that plant water status was unaffected by defoliation (Quentin et al., 2012). It is generally believed that the effect of drought on tree NSCs depends on drought intensity and duration (Mitchell et al., 2014). With the progress of drought, the net photosynthetic rate decrease results in decreased carbohydrate production, but there is relatively little variation in respiration, inducing the consumption of NSC reserves (McDowell and Sevanto, 2010; Galiano et al., 2011). After defoliation, carbohydrate allocation to the new leaves increases, thereby improving individual photosynthetic productivity (Gieger and Thomas, 2002). However, on the other hand, drought always increases biomass allocation to the roots. When drought and defoliation treatment occur at the same time, there will be a trade-off between above-ground and under-ground resource allocation. A previous study reported that the resource limitations under defoliation and drought differentially altered the use of surface water pulses and affected the patterns of fine root allocation in *Populus fremontii* (Snyder and Williams, 2007). Under low-water supply, *Eucalyptus globulus* could compensate for 40% foliage loss by reduction of biomass allocation to coarse roots, mobilization of carbohydrate reserves, and increased ratio of foliage to wood dry mass (Eyles et al., 2009). Defoliation is more likely to reduce NSC levels during drought periods than during non-drought periods (Aguadé et al., 2015). Nevertheless, the combined effects of drought and defoliation on plants are still not fully understood, and more species should be included to study.

As a consequence of climate change, trees with various individual ages or growth sizes are predicted to change in terms of physiological status and carbon reserve substances (Abdul-Hamid and Mencuccini, 2009), which will affect the individual’s response to pest disturbances and drought. Stored NSCs have been proposed to be the key determinants of drought resistance in plants (Galiano et al., 2011). *Pinus Sylvestris* seedlings deplete carbon reserves for root growth under water stress (Ivanov et al., 2019). In *Osteospermum sinuatum*, the production of new leaves after defoliation was found to be dependent on carbon reserves to a great extent (Van der Heyden and Stock, 1996). However, there is still little understanding of the effects of carbon reserve size on plant growth and carbon allocation, particularly under both biotic and abiotic stress conditions (Quentin et al., 2012). The species investigated in the present study, *R. pseudoacacia* and *A. fruticosa*, belong to the Leguminosae family and are fast-growing pioneer species of warm temperate regions in China. Both species are widely used for reforestation due to their high drought tolerance. In the field, their seedlings are always subjected to a wide range of defoliation and water conditions. Under drought stress, *R. pseudoacacia* exhibits more anisohydric behavior than *A. fruticosa* (Li et al., 2019). *R. pseudoacacia* is more susceptible to insect herbivores than *A. fruticosa*. As mentioned above, carbon reserve is essential for plant recovery after defoliation and drought (Galiano et al., 2011), which should also be an important concern in afforestation activities.

In the present study, we investigated the effects of low water availability, artificial defoliation, and plant size on the growth and carbon allocation of two Leguminosae woody species (*R. pseudoacacia* and *A. fruticosa*) in order to investigate how they responded to defoliation and low water availability with different plant sizes. In our study, different plant size resulted in big difference of carbon reserves. We hypothesized that: (1) Defoliation would reduce the effect of low water availability on plant hydraulic parameters; (2) Large seedlings would have more compensatory mechanisms in response to defoliation and drought treatments considering growth, leaf traits and carbon allocation than small seedlings.

## Materials and Methods

### Plant Material and Experimental Design

The experiment was carried out from April to September 2017 at the Fanggan Research Station of Shandong University, Shandong Province, China (36°26′N, 117°27′E). The seeds of *R. pseudoacacia* and *A. fruticosa* were purchased from Qiluyuanyi Seed Company (Linyi, China) and were originally collected from nearby mountains in Shandong Province in the early winter of 2016. The study station was located in warm temperate zone with the mean annual precipitation of 700 ± 100 mm and average temperature of 13 ± 1°C. The soil type is yellow cinnamon soil. The whole experiment was carried out in a greenhouse at the station made up of a steel pipe frame which was covered by a plastic film. During the experimental period, the microclimate in the greenhouse was monitored with HOBO data loggers (U12-012, Onset, Bourne, MA, United States). Mean air temperature was 29.6°C (18.7–36.7°C) during daytime and 20.8°C (10.2–27.5°C) during nighttime, and mean relative humidity was 59.3% (28.2–97.8%) during daytime and 93.6% (56.3–100%) during nighttime.

Two batches of seedlings were grown in advance for our experiment. All seedlings were well watered and protected from grazing by insects, and individuals with similar sizes were selected from each batch for the following treatment. For each species, thirty-two 3 month-old seedlings and thirty-two 1.5 month-old seedlings were selected and randomly assigned to one of the following treatments (four treatments × two plant species, *n* = 8): +WC, high water availability without defoliation; +WDE, high water availability treatment with defoliation; −WC, low water availability without defoliation; and −WDE, low water availability with defoliation. Initial seedling height of large *R. pseudoacacia* seedling was 1.17 ± 0.04 m (*n* = 32) and that of small *R. pseudoacacia* seedling was 0.31 ± 0.02 m (*n* = 32), whereas those of large and small *A. fruticosa* trees were 0.72 ± 0.02 m (*n* = 32) and 0.23 ± 0.01 m (*n* = 32), respectively. The seedlings in the high water availability treatment (+W) were irrigated with 500 mL water every 2 days, whereas those in the low water availability treatment (-W) were irrigated with 500 mL water every 5 days. As the seedlings in the -W treatment exhibited the wilting phenomenon in the experiment, this treatment was referred to as the drought treatment. The seedlings with 50% top down leaf removal were referred to the defoliation treatment (DE), and intact seedlings without leaf removal were served as the control (CK). In both species, defoliation and different water condition treatments were applied on 18th of July, and the plants were harvested on 18th of September (60 days of treatment in total). Eight individuals of each species and treatment were arranged in the experiment.

### Growth and Leaf Trait Measurements

At the end of the treatments, gas-exchange characteristics, including the net photosynthetic rate (*A*), transpiration rate (*E*), intercellular carbon dioxide concentration (*C*_i_), and stomatal conductance (*G*_s_) of fully expanded leaves from five saplings of each species and each treatment were measured using a portable gas exchange measurement system (Li-6800, Li-Cor, Lincoln, NE, United States). These measurements were conducted between 9:00 and 12:00 h. During the measurements, photosynthetically active radiation, temperature, relative humidity, and CO_2_ concentration inside the leaf chamber were controlled at 1,000 μmol m^–2^ s^–1^, 28°C, 50%, and 400 ppm, respectively. After enclosure in the chamber, the leaves were left to acclimate until a constant CO_2_ flux was observed. We checked the parameters of *G*_s_, *C*_i_, and *E* to make sure all of them were positive, and *G*_s_ was mostly between 0 and 1. In addition, we ensured that the ΔCO_2_ range was stable, within 0.5 ppm, and the *A*-value was stable at 1 digit after the decimal point, and it did not increase or decrease in one direction (for up to 5 min).

Seedling basal diameter (BD, at approximately 1 cm above the ground) and height were measured at the end of the experiment. Five to eight seedlings were harvested from each treatment and separated into roots, stems, and leaves. Total leaf area was measured with WinFOLIA Pro 2009a (Regent Instruments, Inc., Quebec, QC, Canada). Thereafter, the samples were oven-dried (30 min at 105°C, followed by 72 h at 75°C) and weighed. Total biomass (TB), leaf mass ratio (LMR), stem mass ratio (SMR), root mass ratio (RMR), root-shoot ratio (R/S), relative growth rate of total biomass (RGR_B_), and net assimilation rate (NAR) were calculated as follows:

TB=RB+SB+LB

$$\text{LMR}=\frac{\text{LB}}{\text{RB}+\text{SB}+\text{LB}}$$

$$\text{SMR}=\frac{\text{SB}}{\text{RB}+\text{SB}+\text{LB}}$$

$$\text{RMR}=\frac{\text{RB}}{\text{RB}+\text{SB}+\text{LB}}$$

$$R/S=\frac{\text{RB}}{\text{SB}+\text{LB}}$$

$${\text{RGR}}_{\text{B}}=\frac{\text{ln}B2-\text{ln}B1}{t}$$

$$\text{NAR}=\frac{\left(\text{ln}T2-\text{ln}T1\right)\left(B2-B1\right)}{\left(T2-T1\right)t}$$

where RB is root biomass, SB is stem biomass, and LB is leaf biomass. *B*1 and *B*2 represent plant total biomass at the beginning and harvest of the treatments, *T*1 and *T*2 represent plant total leaf area at the beginning and harvest of the treatments and *t* represents the duration of treatments (60 days).

### Stem Water Potential and Hydraulic Conductivity

After 2 months of treatment, stem midday (12:00–13:00 h) water potential (Ψ_stem_) was measured with a pressure chamber (1505D-EXP, PMS Instrument, Albany, OR, United States). At the same time, we collected middle stem segments from various individuals of both species for stem hydraulic conductivity measurement. Stem segments were cut under distilled water and rapidly transported to the laboratory with the proximal cut end immersed in water. A second cut was then made under water with a sharp razor blade to remove possible vessel obstructions; the leaves were removed, and scars were sealed with parafilm. Then, the final segments (*ca.* 10 cm in length) were connected to an apparatus with degassed and filtered 0.5 mmol l^–1^ KCl solution. A hydraulic head of 60 cm was used to generate hydrostatic pressure, and the downstream end of the segment was connected to a graduated pipette. The time required for the meniscus in the pipette to cross a certain number of consecutive graduations was recorded. Hydraulic conductivity (*K*_h_, mL mm h^–1^ Pa^–1^) was calculated as *K*_*h*_ = *J*v/(ΔP/ΔL), where *Jv* is the flow rate through the segment (mL h^–1^) and Δ*P/*Δ*L* is the pressure gradient across the segment (Pa mm^–1^). Stem-specific hydraulic conductivity (*K*_s_, mL mm^–1^ h^–1^ Pa^–1^) was calculated as the ratio of *K*_h_ to stem cross sectional area (mm^2^) according to Liu et al. (2015, 2021).

### Non-structural Carbohydrate Analysis

NSCs were analyzed in leaves, stems, and roots of both species after all dried samples were ground in a ball mill. According to previous studies (Cao et al., 2018), NSC were defined here as the sum of starch and soluble sugars. Soluble sugars were extracted twice with 80% ethanol, and starch content was measured after subjecting the solid residue of each sample to a washing step and hydrolysis. The absorbance of the extracts was measured at 620 nm (UV-9000S, Metash, Shanghai, China) after an anthracenone-sulfuric acid reaction. The concentrations of soluble sugars (mg g^–1^) and starch (which were measured and calculated in glucose equivalents) were calculated as the content of measured pool divided by dry weight of the sample. NSC concentration in each organ was calculated by adding the concentrations of soluble sugars and starch.

### Statistical Analysis

The data were first checked for normality and homogeneity. Three-way analysis of variance (ANOVA) was used to detect the main effects and the interactions of species, water availability, and defoliation. One-way ANOVAs followed by Duncan’s multiple comparison were used to test the differences among treatments within species, which were performed at α = 0.05. ANOVAs were performed using IBM SPSS Statistics 23.0 (IBM Corp., Armonk, NY, United States), and the boxplot figures were illustrated using OriginPro 2016 (Originlab Co., Northampton, MA, United States). Redundancy analysis (RDA) and parameter correlations were carried out using the vegan package in R Statistical Software v.4.0.3 (Oksanen et al., 2019; R Core Team, 2020). Linear Mixed Effects Models (LMMs) were used to investigate the effect of “water availability,” “defoliation” and their interaction on plant traits under two plant sizes. Hence, these variables were included as fixed effects in the analyses, “species” was incorporated as a random effect. LMMs were carried out using the nlme package in R Statistical Software v.4.0.3 (Zuur et al., 2009; Pinheiro et al., 2020).

## Results

### Treatment Effects on Plant Growth

**FIGURE 1:**
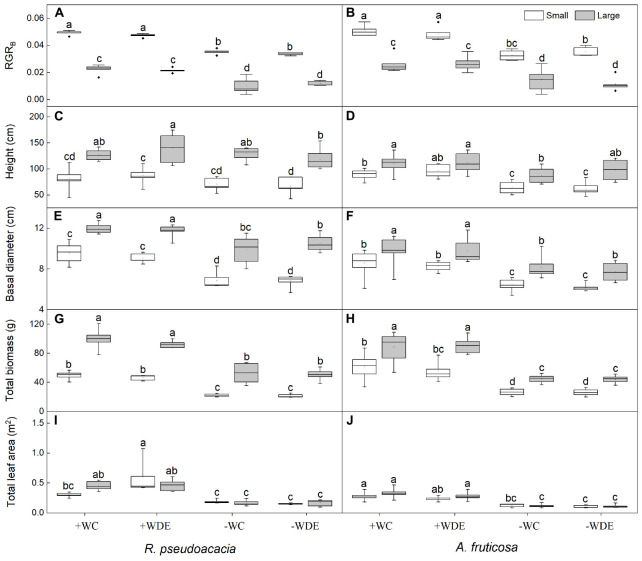
Seedling growth parameters of *Robinia pseudoacacia* **(A,C,E,G,I)** and *Amorpha fruticosa* **(B,D,F,H,J)** under different water availability and defoliation treatments for 60 days of two plant sizes. The values of the boxplot are the mean of 5–8 replicates. Different letters indicate significant differences among different defoliation treatments according to Duncan’s test (*p* < 0.05). +WC, high water availability without defoliation; +WDE, high water availability treatment with defoliation; –WC, low water availability without defoliation; –WDE, low water availability with defoliation; RGR_B_, relative growth rate of total biomass.

**FIGURE 2:**
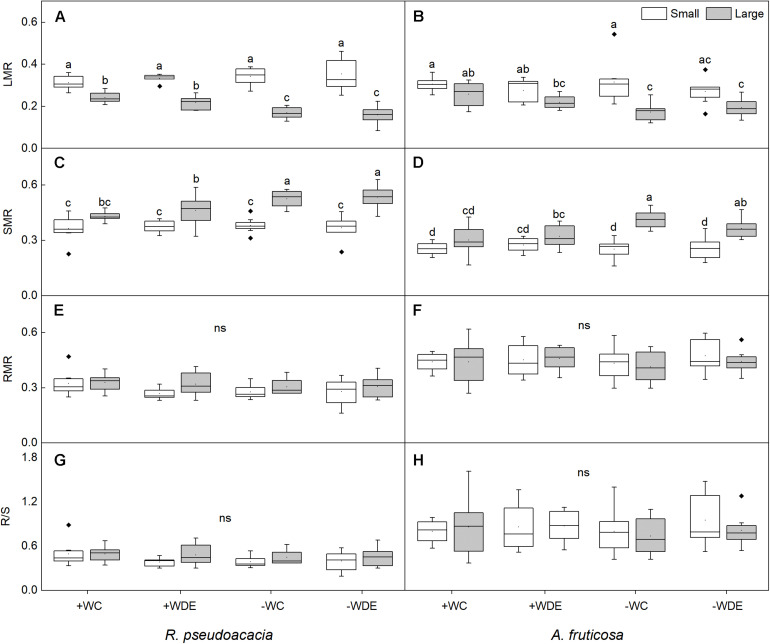
Seedling biomass partitioning parameters of *Robinia pseudoacacia* **(A,C,E,G)** and *Amorpha fruticosa* **(B,D,F,H)** under different water availability and defoliation treatments for 60 days of two plant sizes. The values of the boxplot are the mean of 5–8 replicates. Different letters indicate significant differences among different defoliation treatments according to Duncan’s test (*p* < 0.05). +WC, high water availability without defoliation; +WDE, high water availability treatment with defoliation; –WC, low water availability without defoliation; –WDE, low water availability with defoliation, LMR, leaf mass ratio; SMR, stem mass ratio; RMR, root mass ratio; R/S, root-shoot ratio.

During harvest in September, in *R. pseudoacacia*, the defoliation treatment had no significant effect on seedling growth (Table 1), whereas plant size had a significant impact on seedling growth (Table 1). In all treatments, the height, basal diameter, and biomass of large seedlings were significantly higher than those of small seedlings, but the LMR and RGR_B_ of large seedlings was significantly lower than that of small seedlings (Figures 1, 2A). The RGR_B_ of -W treatments were significantly lower than +W treatments of two plant sizes (Figure 1A). Meanwhile, compared with the + WDE treatment, the −WDE treatment seedlings had 54% lower total biomass in small seedlings and 45% lower total biomass in large seedlings (*p* < 0.05; Figure 1G). The +WDE treatment significantly increased total leaf area of small seedlings, by 54%, when compared with the +WC treatment (*p* < 0.05; Figure 1I). There was no significant difference in SMR among different treatments in small seedlings (Figure 2C). There was no significant difference in R/S and RMR between the plants with large and small seedlings among different treatments (Figures 2E,G).

**TABLE 1:** The proportion of explained variation from water availability (high water availability vs. low water availability), defoliation (defoliation vs. non-defoliation), plant size (small vs. large) and their interactions on 21 measured traits for *R. pseudoacacia* seedlings.

Parameters	W	D	S	W × D	D × S	W × S	W × D × S	R^2^
**Growth measurements**								
*H* (cm)	0.032	<0.001	0.678***	0.018	<0.001	0.004	0.004	0.757
BD (mm)	0.270***	<0.001	0.535***	0.007	0.003	0.0101	<0.001	0.855
TB (g)	0.400***	0.002	0.490***	0.001	< 0.001	0.028***	<0.001	0.933
TLA (m^2^)	0.462***	0.025	< 0.001	0.041	0.027*	0.001	0.037	0.587
RGR_B_	0.190***	<0.001	0.776***	0.001	0.002	0.003	<0.001	0.969
LMR	0.015	<0.001	0.652***	6.360	0.010	0.074***	0.000	0.794
SMR	0.067**	0.005	0.433***	0.002	0.005	0.053	0.000	0.613
RMR	0.028	0.022	0.061	0.017	0.006	<0.001	0.017	0.142
R/S	0.030	0.015	0.046	0.016	0.006	0.000	0.0167	0.127
**Leaf traits and stem hydraulic parameters**								
*A* (μmol m^–2^ s^–1^)	0.928***	<0.001	0.001	<0.001	<0.001	0.001	<0.001	0.930
*E* (mol m^–2^ s^–1^)	0.639***	0.007	0.003	0.009	0.008	0.005	0.011	0.681
*G*_s_ (mol m^–2^ s^–1^)	0.620***	0.000	<0.001	0.000	0.020	0.002	0.020	0.655
NAR (g m^–2^ d^–1^)	0.273***	0.041	0.029	0.112*	0.036	0.003	0.008	0.503
SSHC (*K*_s_, × 10^–3^ mL mm^–1^ h^–1^ Pa^–1^)	0.591***	0.038*	0.078**	0.058**	0.011	0.005	0.015	0.795
SWP (MPa)	0.788***	0.001	0.008	0.041**	0.010	0.013	0.031**	0.893
**Carbon allocation**								
Leaf SS (mg g^–1^)	0.113*	0.003	<0.001	0.010	0.244**	0.006	0.068	0.445
Stem SS(mg g^–1^)	0.013	<0.001	0.008	0.002	0.001	0.005	0.056	0.086
Root SS (mg g^–1^)	0.103*	0.015	0.363***	0.001	0.063*	0.012	0.002	0.560
Leaf ST (mg g^–1^)	0.211	0.001	0.286	0.024	0.001	0.001	0.094	0.618
Stem ST (mg g^–1^)	0.090*	0.021	0.027	0.069*	0.105*	0.156**	0.009**	0.477
Root ST (mg g^–1^)	0.001	0.004	0.017	0.026	0.092	0.020	0.096	0.255

The proportion of explained variance (SSx/SStotal) for each factor and the interactions are indicated. W, water availability treatment; D, defoliation treatment; S, plant size treatment; H, height; BD, basal diameter; TB, total biomass; TLA, total leaf area; RGR_B_, relative growth rate of total biomass; LMR, leaf mass ratio, SMR, stem mass ratio, RMR, root mass ratio, RS, root-shoot ratio; A, the net photosynthetic rate, E, transpiration rate, G_s_, stomatal conductance; NAR, net assimilation rate; SSHC, stem-specific hydraulic conductivity; SWP, stem water potential; Leaf SS, leaf soluble sugar concentration; Stem SS, stem soluble sugar concentration; Root SS, root soluble sugar concentration; Leaf ST, leaf starch concentration; Stem ST, stem starch concentration; Root ST, root starch concentration. *p < 0.05; **p < 0.01; ***p < 0.001. × represents the interaction effect. n = 5–8. Bold values represent significant differences.

In *A. fruticosa*, the defoliation treatment also had no significant effect on seedling growth (Table 2), whereas water availability had a significant impact on seedling growth (Table 2). The RGR_B_ of large seedlings was significantly lower than that of small seedlings (Figure 1A). The RGR_B_ of -W treatments were significantly lower than +W treatments of two plant sizes (Figure 1B). The increase in biomass were 57% less of small seedlings and 49% less of large seedlings in the −WC treatment relative to the +WC treatment, the increase in basal diameter were 25% less of small seedlings and 17% less of large seedlings in the −WC treatment relative to the +WC treatment (*p* < 0.05; Figures 1F,H). The LMR of large seedlings under –WC treatment were significantly lower than those of small seedlings (Figure 2B). There was no significant difference in SMR among different treatments in small seedlings (Figure 2D). There was no significant difference in R/S and RMR between the plants with large and small seedlings among different treatments (Figures 2F,H).

**TABLE 2:** The proportion of explained variation from water availability (high water availability vs. low water availability), defoliation (defoliation vs. non-defoliation), plant size (small vs. large) and their interactions on 21 measured traits for *A. fruticosa* seedlings.

Parameters	W	D	S	W × D	D × S	W × S	W × D × S	R^2^
**Growth measurements**								
*H* (cm)	0.299***	0.003	0.284***	<0.001	<0.001	0.015	0.010	0.624
BD (mm)	0.417***	0.003	0.237***	0.006	0.002	0.002	0.006	0.689
TB (g)	0.558***	0.001	0.232***	<0.001	0.003	0.017*	0.004	0.833
TLA (m^2^)	0.689***	0.020	0.013	0.010	<0.001	0.018	0.000	0.751
RGR_B_	0.266***	0.000	0.613***	0.000	0.001	0.001	0.005	0.873
LMR	0.037	0.024	0.318***	0.009	0.009	0.043*	0.021	0.456
SMR	0.052*	<0.001	0.364***	0.003	0.008	0.083**	<0.001	0.507
RMR	0.002	0.025	0.005	0.000	<0.001	0.007	0.015	0.055
R/S	0.002	0.021	0.003	<0.001	0.002	0.015	0.008	0.053
**Leaf traits and stem hydraulic parameters**								
*A* (μmol m^–2^ s^–1^)	0.839***	0.001	0.003	0.002	0.001	0.006	<0.001	0.445
*E* (mol m^–2^ s^–1^)	0.548***	0.007	0.001	0.014	0.001	<0.001	<0.001	0.531
*G*_s_ (mol m^–2^ s^–1^)	0.864***	<0.001	0.023*	0.001	0.000	0.022*	0.002	0.086
NAR (g m^–2^ d^–1^)	0.295***	<0.001	0.048	0.002	0.011	0.007	0.042	0.406
SSHC (*K*_s_, × 10^–3^ mL mm^–1^ h^–1^ Pa^–1^)	0.559***	0.029	0.029	0.029	0.001	0.059	<0.001	0.618
SWP (MPa)	0.679***	0.008	0.014	0.042*	<0.001	0.038*	0.036*	0.560
**Carbon allocation**								
Leaf SS (mg g^–1^)	0.257**	0.003	0.018	0.026	0.013	0.005	0.007	0.930
Stem SS(mg g^–1^)	0.126**	0.003	0.168**	0.006	0.006	0.167**	0.004	0.681
Root SS (mg g^–1^)	0.234**	0.0349	0.001	0.110*	0.004	0.001	0.001	0.655
Leaf ST (mg g^–1^)	0.219**	0.016	0.005	0.018	<0.001	0.006	0.007	0.795
Stem ST (mg g^–1^)	<0.001	0.002	0.003	0.051	0.001	0.073	0.017	0.893
Root ST (mg g^–1^)	0.009	0.019	0.031	0.012	0.008	0.099	0.002	0.182

The proportion of explained variance (SSx/SStotal) for each factor and the interactions are indicated. W, water availability treatment; D, defoliation treatment; S, plant size treatment; H, height; BD, basal diameter; TB, total biomass; TLA, total leaf area; RGR_B_, relative growth rate of total biomass; LMR, leaf mass ratio, SMR, stem mass ratio, RMR, root mass ratio, RS, root-shoot ratio; A, the net photosynthetic rate, E, transpiration rate, G_s_, stomatal conductance; NAR, net assimilation rate; SSHC, stem-specific hydraulic conductivity; SWP, stem water potential; Leaf SS, leaf soluble sugar concentration; Stem SS, stem soluble sugar concentration; Root SS, root soluble sugar concentration; Leaf ST, leaf starch concentration; Stem ST, stem starch concentration; Root ST, root starch concentration. *p < 0.05; **p < 0.01; ***p < 0.001. × represents the interaction effect. n = 5–8. Bold values represent significant differences.

The linear mixed effects model analysis showed that with the increase of drought stress, the basal diameter and total biomass significantly decreased in large seedlings, but the LMR and total leaf area significantly increased (Supplementary Figure 1). In addition, basal diameter increased with interaction between drought stress and defoliation stress in small seedlings, but not in large seedlings (Supplementary Figure 1). The basal diameter and total biomass were not significantly related to defoliation treatment in both plant sizes (Supplementary Figure 1).

### Treatment Effects on Leaf Traits

**FIGURE 3:**
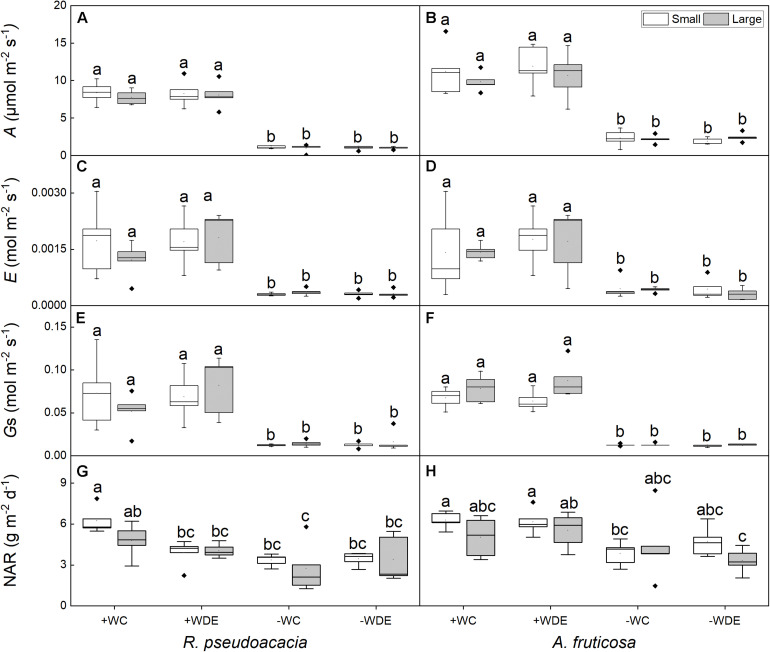
Seedling gas exchange parameters of *Robinia pseudoacacia* **(A,C,E,G)** and *Amorpha fruticosa* **(B,D,F,H)** under various water availability and defoliation treatments for 60 days of two plants sizes. The values of the boxplot are the mean of five replicates. Different letters indicate significant differences among different defoliation and water treatments according to Duncan’s test (*p* < 0.05). +WC, high water availability without defoliation; +WDE, high water availability treatment with defoliation; –WC, low water availability without defoliation; –WDE, low water availability with defoliation, *A*, the net photosynthetic rate; *E*, transpiration rate; *G*_s_, stomatal conductance; NAR, net assimilation rate.

Differences in plant size had no significant effect on leaf traits, and water availability had significant effect on gas exchange parameters in both species (Tables 1, 2). No significant differences were observed in net photosynthetic rate and transpiration rate between small and large seedlings in both species (Figure 3). The seedling gas exchange parameters in plants under +WDE treatment was significantly higher than those in plants under −WDE treatment in both species and in plants of both small and large seedlings (Figure 3). Compared with the + WC treatment, the −WC treatment in *R. pseudoacacia* seedlings had 46% lower NAR in small seedlings and 43% lower NAR in large seedlings (*p* < 0.05; Figure 3G). In *A. fruticosa*, the −WC treatment seedlings had 39% lower NAR in small seedlings compared with the + WC treatment (*p* < 0.05; Figure 3H).

### Treatment Effects on Stem Hydraulic Parameters

**FIGURE 4:**
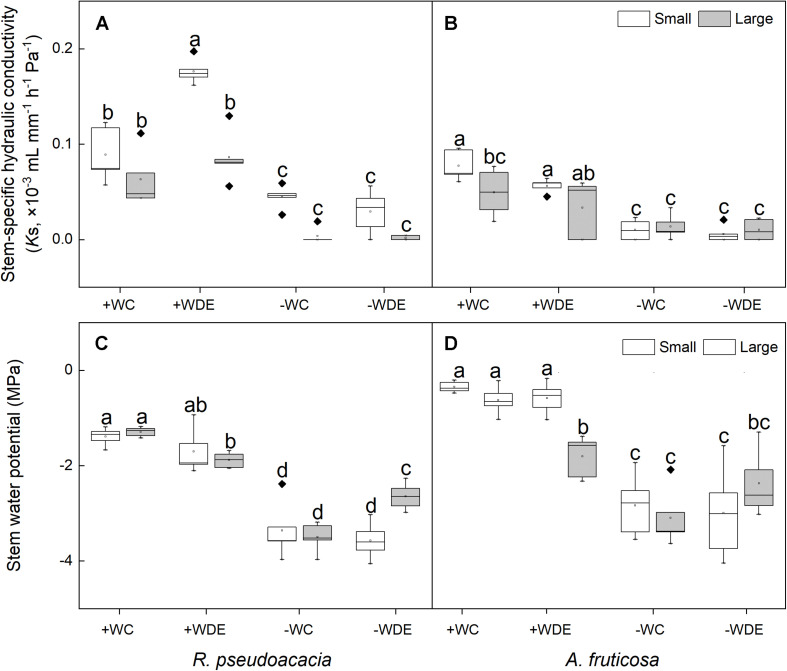
Seedling stem-specific hydraulic conductivity and stem water potential of *Robinia pseudoacacia* **(A,C)** and *Amorpha fruticosa* **(B,D)** under different water availability and defoliation treatments for 60 days of two plant sizes. The values of the boxplot are the mean of five replicates. Different letters indicate significant differences among different defoliation treatments (*p* < 0.05) according to Duncan’s test. +WC, high water availability without defoliation; +WDE, high water availability treatment with defoliation; –WC, low water availability without defoliation; –WDE, low water availability with defoliation.

Stem water potential increased with the interaction between drought stress and defoliation stress of large seedings (Supplementary Figure 2). In *R. pseudoacacia*, water availability, defoliation, and plant size had a significant impact on seedling stem-specific hydraulic conductivity (Table 1). Regardless of defoliation, plants with both small and large sizes had lower water potential and stem-specific hydraulic conductivity under the −W treatments than +W treatments (Figures 4A,C). The stem-specific hydraulic conductivity in the +WDE treatment increased by 98%, when compared to the +CC in small seedlings (*p* < 0.05; Figure 4A). The stem water potential in the −WDE treatment increased by 24%, when compared to the −WC treatment in large seedlings (*p* < 0.05; Figure 4C).

In *A. fruticosa*, the plant water potential in the −WDE treatment decreased significantly, by 412%, when compared in the +WDE treatment in small seedlings (*p* < 0.05; Figure 4D), and it decreased significantly, by 31%, when compared to the +WDE treatment in large seedlings (*p* < 0.05; Figure 4D). There were no significant differences in stem-specific hydraulic conductivity between small seedlings and large seedlings under the +WDE and −WDE treatment (Figure 4B).

### Treatment Effects on Carbon Allocation

The concentrations of soluble sugar and starch in organs were not significantly related to drought stress and defoliation treatment in small seedlings (Supplementary Figure 3). However, in large seedlings, the concentration of stem starch, leaf soluble sugar and leaf starch significantly decreased with the increase of drought stress (Supplementary Figure 3).

**FIGURE 5:**
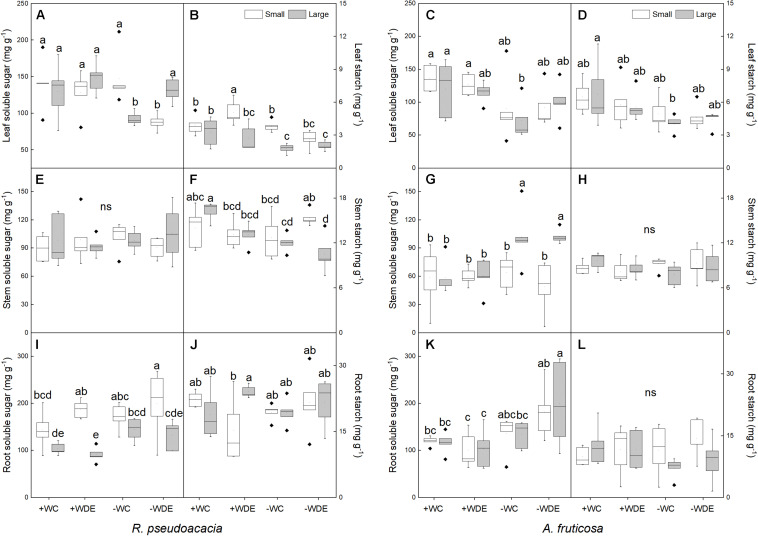
Seedling soluble sugar and starch concentrations in *Robinia pseudoacacia* **(A,B,E,F,I,J)** and *Amorpha fruticosa* **(C,D,G,H,K,L)** seedlings of two plant sizes under different defoliation and water availability treatments on day 60. The values of the boxplot are the mean of five replicates. Different letters indicate significant differences among different defoliation treatments (*p* < 0.05) according to Duncan’s test. +WC, high water availability without defoliation; +WDE, high water availability treatment with defoliation; –WC, low water availability without defoliation; –WDE, low water availability with defoliation.

In *R. pseudoacacia*, defoliation treatment had no significant effect on carbon allocation (Table 1). −WC treatment significantly decreased leaf soluble sugar concentration, by 29%, and starch concentration, by 46%, when compared with the +WC treatment in large seedlings (*p* < 0.05; Figures 5A,B). Defoliation and plant size had interactive effects on leaf soluble sugar concentration (Table 1). Compared with the +WDE treatment, the −WDE treatment significantly decreased leaf soluble sugar concentration, by 32%, in small seedlings (*p* < 0.05; Figure 5A). Small seedlings had higher root soluble sugar concentrations than those of large plants (Figure 5I). Meanwhile, compared with +WC treatment, the −WC treatment also significantly decreased stem starch concentrations in large plants, by 26%, (*p* < 0.05; Figure 5F), but not in small plants (Figure 5F).

In *A. fruticosa*, defoliation treatment also had no significant effect on carbon allocation (Table 2). On the other hand, water supply had a significant impact on carbon allocation (Table 2). In large seedlings, −WC treatment significantly decreased leaf soluble sugar concentration and leaf starch concentration by 40%, when compared to +WC treatment (*p* < 0.05; Figures 5C,D), and −WDE treatment significantly increased stem and root soluble sugar concentrations, by 69 and 92%, respectively, when compared with the +WDE treatment (*p* < 0.05; Figures 5G,K). Under conditions of low water content, large seedlings had higher stem soluble sugar concentration than those of small plants (Figure 5G). Water availability and defoliation had an interactive effect on root soluble sugar concentration (Table 2). The −WDE treatment significantly increased root soluble sugar concentrations by 59% when compared with the −WCK treatment in large seedlings (*p* < 0.05; Figure 5K).

### Relationships and Trade-Offs Among Plant Traits

**FIGURE 6:**
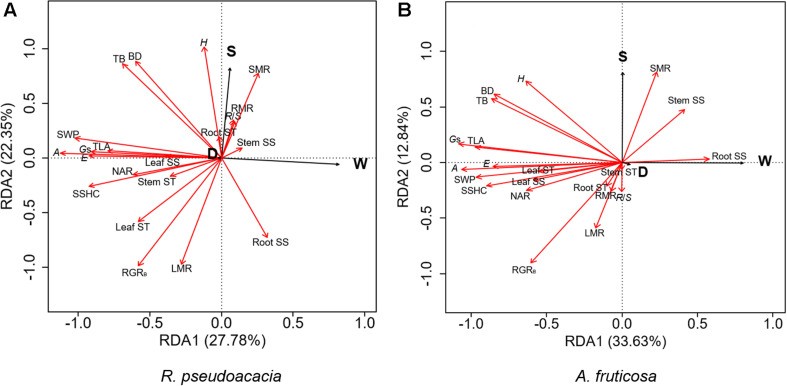
Redundancy analysis (RDA) of the effects of defoliation (D), water availability (W), and plant size (S) on plant traits of *Robinia pseudoacacia* **(A)** and *Amorpha fruticosa* **(B)**. The black line vectors represent treatment factors (defoliation, water supply, and plant size), and the red line vectors represent plant traits. *H*, height; BD, basal diameter; TB, total biomass; TLA, total leaf area; RGR_B_, relative growth rate of total biomass; LMR, leaf mass ratio, SMR, stem mass ratio, RMR, root mass ratio, RS, root-shoot ratio; *A*, the net photosynthetic rate, *E*, transpiration rate, *G*_s_, stomatal conductance; NAR, net assimilation rate; SSHC, stem-specific hydraulic conductivity; SWP, stem water potential; Leaf SS, leaf soluble sugar concentration; Stem SS, stem soluble sugar concentration; Root SS, root soluble sugar concentration; Leaf ST, leaf starch concentration; Stem ST, stem starch concentration; Root ST, root starch concentration.

RDA was performed for two species, respectively (Figure 6). The first two axes explained 50.13% of the variation in *R. pseudoacacia* (Figure 6A), and 46.47% of the variation in *A. fruticosa* (Figure 6B). In both species, the gas exchange parameters were positively correlated with hydraulic parameters, while the root soluble sugar concentration was negatively correlated with the leaf soluble sugar concentration. The LMR was positively correlated with RGR_B_ in both species (Figure 6). The effect of water availability (W) and plant size (S) were more significant than defoliation (D) on plant traits. Water availability treatment was negatively correlated with gas exchange parameters and hydraulic parameters. Plant size was positively correlated with plant height and SMR, but negatively correlated with LMR.

## Discussion

### Influence of Water Availability on Seedling Responses to Defoliation

Previous studies have reported that defoliation could increase net photosynthetic rate of plant seedlings (Quentin et al., 2012; Barry and Pinkard, 2013). However, no increase in photosynthesis was observed after defoliation for 60 days in our research. Some studies have also suggested that defoliation does not significantly increase photosynthesis. For example, in a study with white birch and balsam poplar, defoliation did not significantly increase photosynthesis in the remaining leaves (Man et al., 2013). In *Larix leptolepis* and *Pinus resinosa* seedlings, defoliation was also found had minimal effects on photosynthesis (Kruger et al., 1998). Photosynthetic responses to defoliation are thought to depend on water availability (Pinkard et al., 2011). In the present study, under high water availability treatment, no increase in photosynthesis was observed after defoliation, and defoliation did not result in a loss of total biomass in defoliated seedlings (Tables 1, 2 and Supplementary Figure 1), which may be ascribed to species-specific responses, or we missed the photosynthetic rise during the 60 days’ treatment. In addition, the results of the linear mixed effects model illustrated that the stem water potential was sensitive to the interaction between the defoliation treatment and water availability treatment, which was consistent with the results of three-way analysis of variance (Tables 1, 2 and Supplementary Figure 2). We found that the defoliation treatment and water availability treatment had an interactive effect on the water potential of *R. pseudoacacia* (Table 1). In large *R. pseudoacacia* seedlings, −WDE treatment significantly increased the stem water potential compared with −WC treatment (Figure 4), which showed that defoliation alleviated cavitation under low water availability because the leaf transpiration pull is lessened. The vulnerability of plants to cavitation was low, which protected the trees and minimized the damage caused by xylem embolism (Borchert and Pockman, 2005).

As osmotically active compounds, soluble sugars perform specific protective functions in plants under water stress (Ivanov et al., 2019). Studies have shown that to overcome negative carbon balance, defoliation treatment increases sugar allocation to the leaves (Eyles et al., 2009), while drought increases sugar allocation to the roots (Galvez et al., 2011; Ivanov et al., 2019). In the present study, defoliation did not affect carbon allocation in either species, but drought significantly affected carbon allocation of the seedlings after 2 months. Interestingly, when defoliation and low water availability occurred simultaneously, we found that they interacted to increase root soluble sugar concentrations in *A. fruticosa.* In small seedlings of *A. fruticosa*, −WDE treatment significantly increased root soluble sugar concentration compared with +WDE treatment, but there was no significant difference in root soluble sugar concentration between +WC treatment and +WDE treatment (Figure 5), indicating that compared to defoliation, water availability had a greater influence on plant carbon allocation in *A. fruticosa* seedlings in our experiment.

### Influence of Plant Size on Seedling Responses to Drought and Defoliation

Researches had shown that both *R. pseudoacacia* and *A. fruticosa* were pioneer species with fast growth rates and commonly used for vegetation restoration (Wang and Zhou, 2000; Dehaan et al., 2006; Li et al., 2019). Moreover, in the present study, we observed that the RGR_B_ of the small seedlings was significantly higher than that of the large seedlings in both species (Figure 1), which showed that small seedlings accumulated more biomass than large seedlings during growth stage. Meanwhile, we found that LMR was positively correlated with RGR_B_ (Figure 6). Previous studies show that LMR is an important determinant of relative growth rate (Simane et al., 1993; Warren and Adams, 2005; Imada et al., 2010). The results showed that plant size could significantly affect biomass allocation to leaves and relative growth rate. However, low water availability treatment significantly decreased plant total biomass and RGR_B_ regardless of plant size in both species, and these results were consistent with those of previous studies (Prieto et al., 2009; Li et al., 2019). This may be related to the weak photosynthetic capacity, severe hydraulic embolism, and carbon allocation change in seedlings under drought conditions compared to those under well water conditions (Quentin et al., 2012; Duan et al., 2013; Salmon et al., 2019). Under low water availability treatment, leaf mass ratio of large seedlings of both species was significantly lower than that under high water availability treatment, but this was not observed in small seedlings. This indicated that the investment of plants in leaf biomass was significantly reduced as a consequence of drought, which may be related to growth potential constraints and aboveground plasticity (Maseda and Fernández, 2016). Compared to high water availability treatment, low water availability treatment significantly decreased plant photosynthetic rate, stomatal conductance, and transpiration rate (Ohashi et al., 2006). In the present study, we found that compared to non-drought conditions, drought significantly decreased plant gas exchange, but we did not observe any difference in gas exchange parameters as a consequence of different plant sizes (Figure 3). When plants are under drought stress, stomatal closure, or shrinkage leads to a decrease in stomatal conductance compared to that in non-stressed plants, and in addition, the amount of CO_2_ absorbed by the plant decreases, resulting in a decrease in photosynthetic rate (Rancourt et al., 2015). In addition, the results of the linear mixed effects model illustrated that NAR of small seedlings was sensitive to defoliation treatment, water availability treatment, and the interaction of defoliation and water availability treatment (Supplementary Figure 2), but the NAR of large seedlings was relatively stable under defoliation and water availability. This showed that large seedlings have stronger ability to deal with pest damage and drought. We also found that the NAR of −WC treatment was significantly lower than +WC treatment in the small seedlings, but the large seedlings of −WC treatment did not significantly decrease the NAR compared with control in *A. fruticosa* (Figure 3). For *R. pseudoacacia*, the NAR of −WDE treatment was significantly lower than +WC treatment in the small seedlings, but not in large seedlings (Figure 3). The results also showed that larger seedlings had a greater potential to survive under various stress (Delagrange et al., 2004; Issifu et al., 2015). However, we observed that in the high water availability treatments, the total leaf area and stem-specific hydraulic conductivity of small seedlings of *R. pseudoacacia* increased significantly in defoliated seedlings compared with those in non-defoliated seedlings, but these changes were not observed in large seedlings (Figure 4A). This may be related to the fact that small seedlings were more sensitive to defoliation treatment.

As an important response strategy, plants will rely on stored carbon reserves under drought (Hartmann et al., 2013). Sugars and starch are the main components of mobile carbon pools in plants (Ivanov et al., 2019). In this study, we found that large *R. pseudoacacia* seedlings reduced stem starch concentration in −WC treatment compared to +WC treatment, and stem soluble sugar concentration remained unchanged, but this was not exhibited in small *R. pseudoacacia* seedlings (Figure 5). These results indicated the corresponding conversion of starch to soluble sugars in large carbon reserve seedlings (Tomasella et al., 2019). Starch functions as a storage compound and could be depleted in plants under low water availability treatment (Martínez-Vilalta et al., 2016). Seedlings with large carbon reserves can mobilize these reserves for osmotic adjustment in order to maintain normal physiological activities under drought stress (Hasibeder et al., 2015), but small seedlings do not have sufficient carbon reserves for carbon dynamics transformation to maintain osmotic adjustment.

### Influence of Drought, Defoliation, and Plant Size on the Response Strategies of the Two Species

Previous studies on the influence of defoliation on plant seedling growth have shown reduced plant growth or no significant change in plant growth following partial defoliation (Pinkard et al., 2006; Quentin et al., 2011; Barry and Pinkard, 2013). In the present study, there were no significant changes in plant height, basal diameter, and total biomass following the defoliation treatment of two plant sizes in both species (Figure 1), suggesting that plants use a range of response strategies to compensate for the impacts of defoliation. We also found that in the +W treatment, defoliation significantly increased stem-specific hydraulic conductivity in small seedlings of *R. pseudoacacia*, but this was not observed in *A. fruticosa* (Figure 4). This may be related to the fact that *R. pseudoacacia* is an anisohydric species (Li et al., 2019); after defoliation, anisohydric species adopt an aggressive water consumption strategy to improve their osmotic adjustment ability and maintain cell swelling pressure (Rosas et al., 2019). In *A. fruticosa*, root soluble sugar concentration increased in response to drought. This increase was concurrent with declines in leaf soluble sugar concentration, suggesting a potential trade-off between allocation of photoassimilates to roots vs. leaves during drought, which is also confirmed by the RDA analysis (Figure 6). This shows that soluble sugar is an important metabolic substrate and osmoregulatory compound under drought (Dietze et al., 2014), and the increase in the root soluble sugar concentration is benefit for increasing root productivity and water absorption capacity (Gieger and Thomas, 2002). Additionally, our study also found that defoliation had no significant effect on carbon allocation in small seedlings, which was consistent with the results of the linear mixed effects model (Supplementary Figure 3), but compared to high water availability treatment, low water availability treatment significantly decreased leaf starch concentration in the two species. The reduction in carbon storage was mainly because of the reduction in photosynthetic carbon assimilation, which was a consequence of closed stomata (Galiano et al., 2011).

## Conclusion

In this experiment, we highlight the importance of plants sizes in seedling responses to defoliation and water availability regimes. There were no significant changes in plant height, basal diameter, and total biomass following the defoliation treatment of two plant sizes. Compared to high water availability treatment, low water availability treatment significantly decreased plant gas exchange parameters. Defoliation would reduce the effect of low water availability in large seedlings on hydraulic parameters. The relative growth rate of large seedlings was significantly lower than that of small seedlings. Large *R. pseudoacacia* seedlings had a strong ability to deal with defoliation treatment, and large *A. fruticosa* seedlings had a strong ability to deal with low water availability treatment. Large seedlings can mobilize their reserves for osmotic adjustment. The *R. pseudoacacia* and *A. fruticosa* seedlings were able to quickly recover from defoliation because of their rapid growth, but were affected more severely by drought in this study. Our findings could provide a basis for the evaluation of forest dynamics under global climate change, and make a contribution to the theoretical and practical researches of vegetation restoration. Considering the trees with large plant sizes have stronger ability to deal with pest damage and drought, optimizing the forest age structure could effectively avoid the rapid forest decline in the events of combined drought and biotic attack.

## Data Availability Statement

The raw data supporting the conclusions of this article will be made available by the authors, without undue reservation.

## Author Contributions

NW conducted the experiment, wrote the body of the manuscript, and performed sample preparations, and laboratory and data analyses. ND and HW set up the experimental design and provided funding. MZ, XS, and XP performed the experiments. QL, XL, SY, and HS conducted analyses. PF, QG, YW, LY, and RW contributed in editing the manuscript. All authors contributed to the article and approved the submitted version.

## Conflict of Interest

The authors declare that the research was conducted in the absence of any commercial or financial relationships that could be construed as a potential conflict of interest.
