# Supplementary material for: Plant Size Plays an Important Role in Plant Responses to Low Water Availability and Defoliation in Two Woody Leguminosae Species
Source: Front Plant Sci. 2021 Apr 9;12:643143. doi: 10.3389/fpls.2021.643143 (PMC8062765; doi:10.3389/fpls.2021.643143)
Supplement: Supplementary file 1 [file Data_Sheet_1.docx]

**Supplementary materials**

**
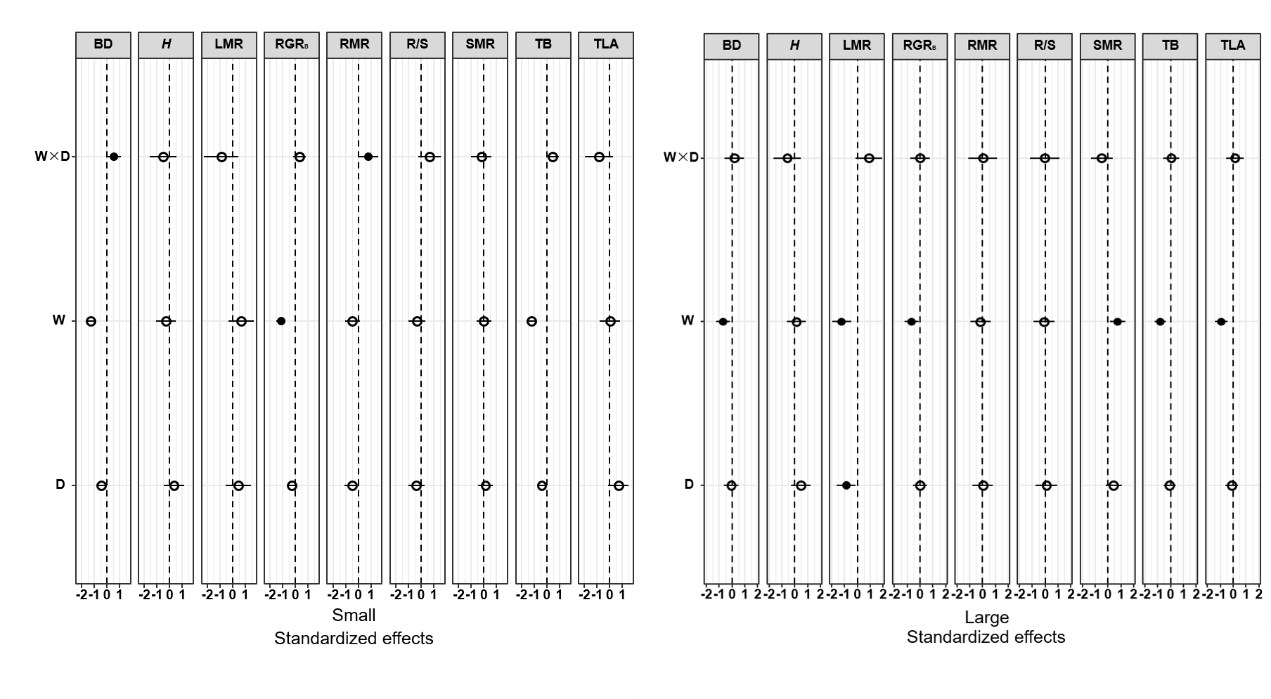
**

**Fig. S1** Estimates of the coefficients (± 2 × SE) for seedling growth parameters of two plant sizes under different defoliation and water availability treatments on day 60. Solid symbols indicate parameters significantly different from zero (*p* < 0.05), and hollow symbols indicate no significant difference from zero (*p* > 0.05). W, water availability treatment; D, defoliation treatment; BD, basal diameter; *H*, height; LMR, leaf mass ratio; RGR_B_, relative growth rate of total biomass; RMR, root mass ratio; RS, root-shoot ratio; SMR, stem mass ratio; TB, total biomass; TLA, total leaf area. × represents the interaction effect.

**
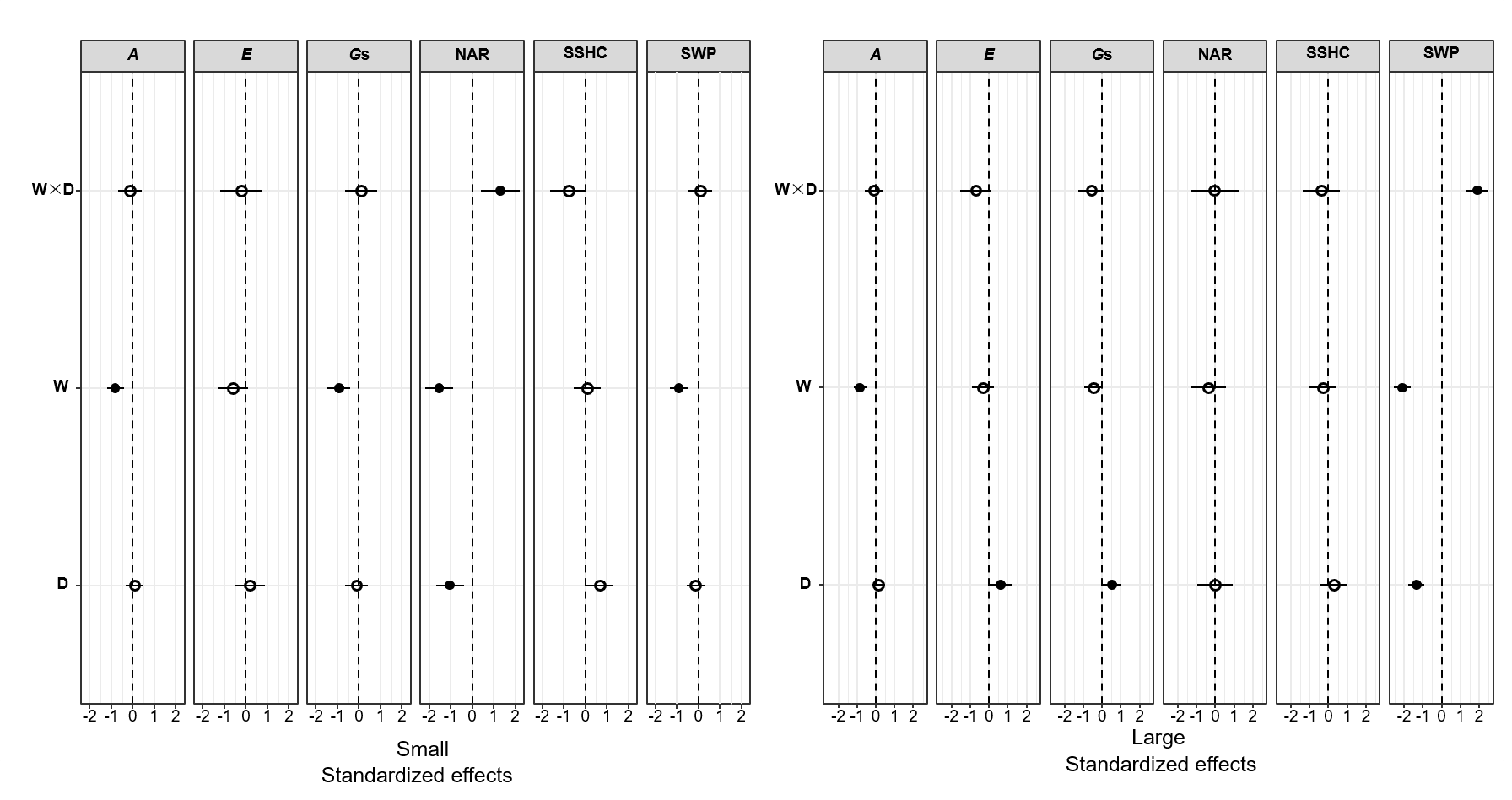
**

**Fig. S2** Estimates of the coefficients (± 2 × SE) of seedling leaf traits and hydraulic parameters of two plant sizes under different defoliation and water availability treatments on day 60. Solid symbols indicate parameters significantly different from zero (*p* < 0.05), and hollow symbols indicate no significant difference from zero (*p* > 0.05). W, water availability treatment; D, defoliation treatment; *A*, net photosynthetic rate; Chl *a/b*, Chlorophyll *a/b*; *E*, transpiration rate, *G*_s,_ stomatal conductance; NAR, net assimilation rate; SSHC, stem-specific hydraulic conductivity; SWP, stem water potential; Chl _total_, total chlorophyll concentration. × represents the interaction effect.


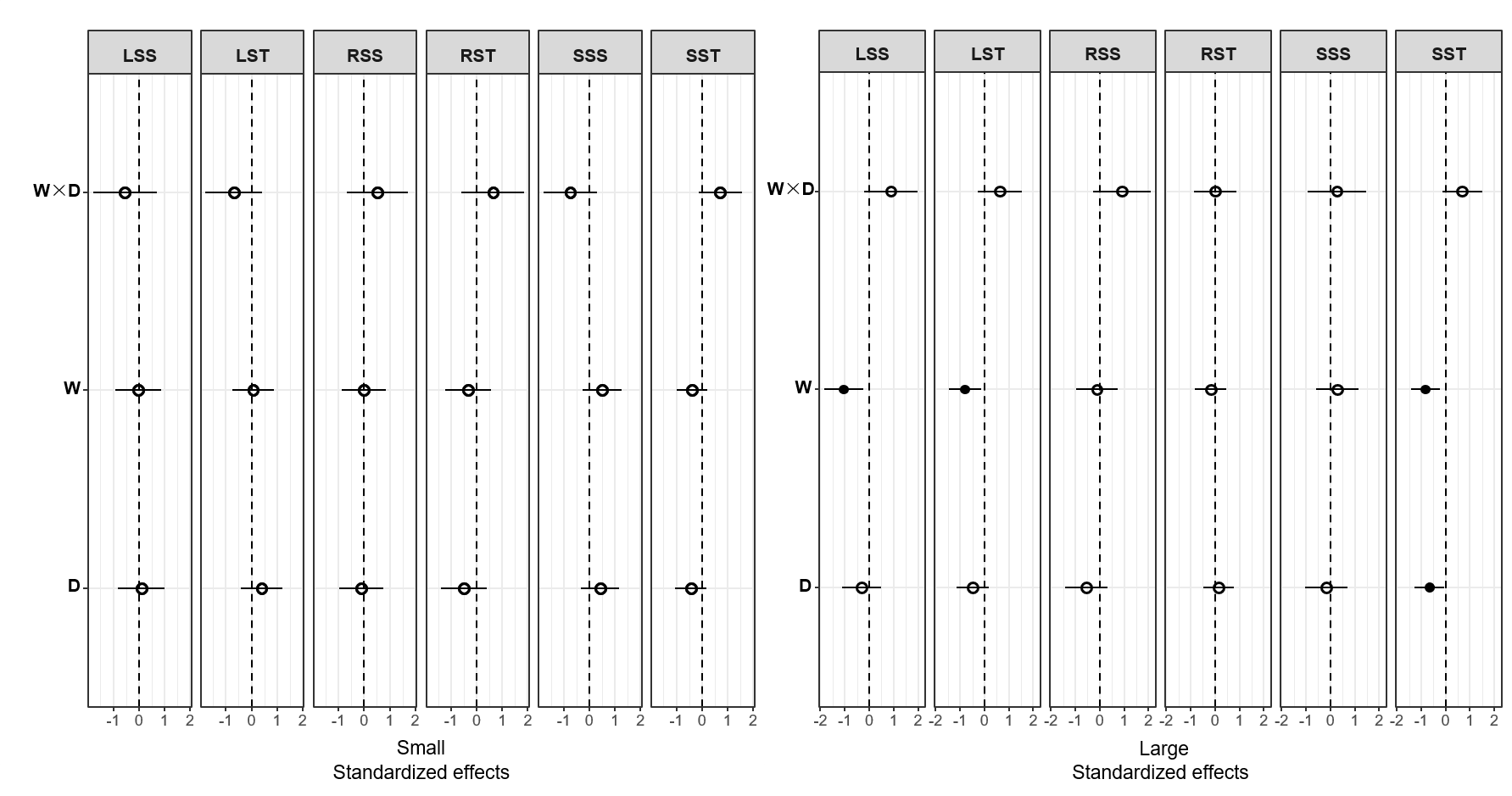


**Fig. S3** Estimates of the coefficients (± 2 × SE) for seedling soluble sugar and starch concentration of two plant sizes under different defoliation and water availability treatments on day 60. Solid symbols indicate parameters significantly different from zero (*p* < 0.05), and hollow symbols indicate no significant difference from zero (*p* > 0.05). W, water availability treatment; D, defoliation treatment; LSS, leaf soluble sugar concentration; LST, leaf starch concentration; RSS, root soluble sugar concentration; RST, root starch concentration; SSS, stem soluble sugar concentration; SST, stem starch concentration. × represents the interaction effect.
